# Vade Mecum of Equine Anatomy

**Published:** 1881-04

**Authors:** 


Vade Mecum of Equine Anatomy, for the Use of Advanced Students and Veterinary Surgeons. By A. Liautard, M.D., D.V.S. New York: W. R. Jenkins. 1879.

This work hardly comes up to that of McBride, though it is not entirely without merit.

